# Factors associated with non-adherence to angiotensin-converting enzyme inhibitors and angiotensin receptor blockers in older patients with peripheral arterial disease

**DOI:** 10.3389/fphar.2023.1199669

**Published:** 2023-08-10

**Authors:** Martin Wawruch, Miriam Petrova, Tomas Tesar, Jan Murin, Patricia Schnorrerova, Martina Paduchova, Denisa Celovska, Beata Havelkova, Michal Trnka, Sofa D. Alfian, Emma Aarnio

**Affiliations:** ^1^ Institute of Pharmacology and Clinical Pharmacology, Faculty of Medicine, Comenius University, Bratislava, Slovakia; ^2^ Department of Organisation and Management of Pharmacy, Faculty of Pharmacy, Comenius University, Bratislava, Slovakia; ^3^ 1st Department of Internal Medicine, Faculty of Medicine, Comenius University, Bratislava, Slovakia; ^4^ Department of Angiology, Health Centre, Trnava, Slovakia; ^5^ General Health Insurance Company, Bratislava, Slovakia; ^6^ Institute of Medical Physics, Biophysics, Informatics and Telemedicine, Faculty of Medicine, Comenius University, Bratislava, Slovakia; ^7^ Department of Pharmacology and Clinical Pharmacy, Faculty of Pharmacy, Universitas Padjadjaran, Bandung, Indonesia; ^8^ Center of Excellence in Higher Education for Pharmaceutical Care Innovation, Universitas Padjadjaran, Bandung, Indonesia; ^9^ School of Pharmacy, University of Eastern Finland, Kuopio, Finland

**Keywords:** peripheral arterial disease, non-adherence, non-persistence, polypharmacy, new user, dementia, general practitioner, Anxiety disorder

## Abstract

**Introduction:** As in other chronic conditions, medication adherence is important in the treatment of peripheral arterial disease (PAD). Our study aimed at a) analysing non-adherence to angiotensin-converting enzyme inhibitors (ACEIs) and angiotensin receptor blockers (ARBs) in groups of older ACEI and ARB users with PAD, and b) identifying characteristics associated with non-adherence.

**Methods:** We focused on the implementation phase of adherence (i.e., after treatment initiation and before possible discontinuation of treatment). The study cohort included ACEI/ARB users aged ≥65 years in whom PAD was newly diagnosed during 2012. Non-adherence was defined as Proportion of Days Covered (PDC) < 80%.

**Results:** Among 7,080 ACEI/ARB users (6,578 ACEI and 502 ARB users), there was no significant difference in the overall proportion of non-adherent patients between ACEI and ARB users (13.9% and 15.3%, respectively). There were differences in factors associated with non-adherence between the groups of persistent and non-persistent (i.e., discontinued treatment at some point during follow-up) ACEI and ARB users. Increasing age, dementia and bronchial asthma were associated with non-adherence in persistent ACEI users. General practitioner as index prescriber was associated with adherence in the groups of non-persistent ACEI users and persistent ARB users.

**Conclusion:** Identified factors associated with non-adherence may help in determining the groups of patients who require increased attention.

## 1 Introduction

Peripheral arterial disease (PAD) represents the third most important cause of atherosclerotic morbidity following stroke and coronary heart disease (Criqui et al., 2021). In 2010, 202 million people suffered from PAD globally (Fowkes et al., 2013). The prevalence of PAD increases with age and is similar among men and women. In high-income countries, the prevalence of PAD at the age of 45–49 years was 5.3% among women and 5.4% among men, and at the age of 85–89 years, corresponding figures were 18.4% and 18.8%, respectively. Between the years 2000 and 2010, the number of patients with PAD increased by 13.1% in high income countries and by 28.7% in low- and middle-income countries, resulting in 69.7% of patients with PAD living in low- or middle-income countries in 2010 (Fowkes et al., 2013). PAD is associated with annual mortality rate of 4%–6%. Despite its prevalence and clinical relevance, PAD is underappreciated by physicians and patients (Dua and Lee, 2016; Malyar et al., 2016; Criqui et al., 2021).

PAD is defined as a progressive disorder accompanied with stenosis and/or occlusion of large and medium-sized arteries, other than those that supply the heart or brain (Shu and Santulli, 2018). This disease may affect extracranial carotid and vertebral arteries, upper and lower extremity arteries, mesenteric arteries and renal arteries (Aboyans et al., 2018). In our study, PAD refers to atherosclerotic disease of arteries of lower limbs. It represents a strong risk factor for major adverse cardiac events (myocardial infarction (MI), stroke, cardiovascular (CV) death) and major adverse limb events (acute limb ischemia, critical limb ischemia, major amputations). Smoking, diabetes mellitus, dyslipidaemia, arterial hypertension, and chronic kidney disease are major modifiable risk factors of PAD (Bonaca and Creager, 2015; Firnhaber and Powell, 2019; Bevan and White Solaru, 2020; Criqui et al., 2021; Gupta and Patel, 2022). Conservative treatment of PAD includes smoking cessation, exercise training in patients with intermittent claudication and administration of secondary preventive medications: statins, antiplatelet agents and antihypertensive medications. Calcium channel blockers, diuretics, beta-blockers, angiotensin-converting enzyme inhibitors (ACEIs), and angiotensin receptor blockers (ARBs) represent suitable classes of antihypertensive medications in PAD patients (Gerhard-Herman et al., 2017; Aboyans et al., 2018; Bevan and White Solaru, 2020; Golledge, 2022). However, according to European and American guidelines, ACEIs and ARBs should be considered as first-line antihypertensive medications (Gerhard-Herman et al., 2017; Aboyans et al., 2018). This recommendation is based on the results of the Heart Outcomes Prevention Trial (HOPE) and the Ongoing Telmisartan Alone and in Combination with Ramipril Global Endpoint Trial (ONTARGET) (Yusuf et al., 2000; Yusuf et al., 2008). In these trials, ACEIs and ARBs significantly reduced CV events in patients with PAD.

Adherence to medications represents a basic precondition of successful treatment of PAD. Adherence consists of three phases: initiation, implementation, and persistence. Initiation represents taking the first dose of a prescribed medication. Implementation reflects the extent to which a patient´s dosing regimen corresponds to that recommended by the physician. Persistence represents the time between initiation and the last dose before discontinuation (stopping treatment) (Vrijens et al., 2012; De Geest et al., 2018).

Sung et al. (2009) and Cui et al. (2020) reported differences in patients´ adherence to antihypertensive therapy associated with the use of different antihypertensive medications. Based on these findings, our study compared treatment adherence between ACEI and ARB users focusing on the implementation phase of medication adherence. The aims of our study were: a) to analyse non-adherence to ACEI/ARB treatment separately among older persistent and non-persistent ACEI and ARB users with newly diagnosed PAD; and b) to identify patient- and medication-related characteristics associated with non-adherence in these groups of patients. Patients were divided into two groups based on their persistence status to see if there are differences between persistent and non-persistent patients already during the implementation phase. To the best of our knowledge, there is no similar study focused on non-adherence to ACEI/ARB treatment in older PAD patients.

## 2 Materials and methods

### 2.1 Database and study population

The data for our retrospective register-based study were collected from the database of the General Health Insurance Company, the largest health insurance provider in Slovakia which covers approximately 63% of the population. From this database, patients in whom PAD was newly diagnosed between 1 January and 31 December 2012 were identified. From them, patients aged ≥65 years treated with ACEIs or ARBs were selected into the study cohort. The derivation of the study cohort is described in our previous manuscript (Wawruch et al., 2022).

### 2.2 Analysis of non-adherence to ACEI/ARB treatment

Our study focused on the implementation phase of medication adherence. Proportion of Days Covered (PDC) represents a method to evaluate this phase in this type of register-based data (Karve et al., 2009; Giardini et al., 2016). PDC was calculated as a ratio of the number of days covered by adequate number of tablets of ACEIs/ARBs and the number of days of the follow-up period during which a patient was persistent with ACEI/ARB treatment. Once daily dosing was assumed for ACEIs and ARBs used in patients of our study cohort. For persistent patients, the number of all days of the follow-up period represented the denominator of PDC index. On the other hand, in non-persistent patients, only the number of days during which the patient was persistent with ACEI/ARB treatment was used as the denominator (Alfian et al., 2018). This restriction was introduced in order to avoid overestimation of non-adherence in these patients caused by non-persistence with treatment. Patients with PDC<80% were considered as non-adherent (Karve et al., 2009). Non-persistence was identified based on the presence of at least 6-month tablet-free gap after the estimated period covered by the last prescription.

### 2.3 Factors associated with non-adherence to ACEI/ARB treatment

Factors associated with the likelihood of non-adherence were analysed separately in persistent and non-persistent patients. The same patient- and medication-related characteristics as those included in our previous study on non-persistence (Wawruch et al., 2022) were analysed as factors potentially associated with non-adherence in this study. The data on these characteristics were assembled at the time of inclusion into the study, except for history of CV events which covered the period of 5 years before the index date of the study (the date of the first dispensation of ACEI/ARB after the diagnosis of PAD). Patients in whom ACEI/ARB treatment was initiated after the diagnosis of PAD were considered as new users, whereas those in whom ACEI/ARB treatment was initiated before PAD diagnosis represented the group of prevalent users.

### 2.4 Statistical analysis

Continuous variables were characterised as means ± standard deviations (SD) and categorical variables as frequencies and percentages.

Categorical variables were compared between the two groups using the χ^2^-test. The Fisher exact test was applied in the case when the expected count was less than five in ≥20% of cells of the contingency table. The Mann-Whitney U test was applied to compare continuous variables between the two groups. The reason for the use of this non-parametric test was the non-Gaussian distribution of evaluated variables. The normality of the distribution was analysed with the Kolmogorov-Smirnov test.

The most important characteristics associated with the probability of non-adherence were identified with binary logistic regression. The method of forward conditional was applied in this model. Results are presented as odds ratios with 95% confidence intervals (Newman, 2001).

All statistical tests were carried out at the significance level of *α* = 0.05. The statistical software IBM SPSS for Windows, version 29, was used (IBM SPSS Inc., Armonk, NY, USA).

### 2.5 Sensitivity analysis

Since the 5-year follow-up period is relatively long, we identified factors associated with non-adherence in the model with a shorter 3-year follow-up period.

## 3 Results

The baseline characteristics of the study cohort are described in Table 1A and Table 2. The study cohort of 7,080 patients included 6,578 (92.9%) ACEI and 502 (7.1%) ARB users. There was no significant difference in the proportions of non-persistent patients between ACEI and ARB users (23.0% and 25.9%, respectively; *p* = 0.136 according to the χ^2^-test). We also did not find any significant difference in the overall proportions of non-adherent patients between ACEI and ARB users (13.9% and 15.3%, respectively; *p* = 0.379 according to the χ^2^-test).

**TABLE 1:** Baseline characteristics of the cohort of ACEI users.

Factor	ACEI users (n = 6,578)						
	All ACEI users (n = 6,578)	Persistent (n = 5,066; 77.0%)			Non-persistent (n = 1,512; 23.0%)		
		Adherent (n = 4,449; 87.8%)	Non-adherent (n = 617; 12.2%)	p	Adherent (n = 1,213; 80.2%)	Non-adherent (n = 299; 19.8%)	p
*Socio-demographic characteristics*							
Age	75.2 ± 6.8	75.5 ± 6.9	76.6 ± 7.1	<0.001**	74.2 ± 6.2	73.4 ± 6.2	0.051**
Female sex	3,703 (56.3)	2,508 (56.4)	324 (52.5)	0.070	702 (57.9)	169 (56.5)	0.672
University education	467 (7.1)	315 (7.1)	40 (6.5)	0.586	90 (7.4)	22 (7.4)	0.971
Employed patients	331 (5.0)	226 (5.1)	23 (3.7)	0.145	62 (5.1)	20 (6.7)	0.281
*History of CV events* ^a^							
History of ischemic stroke	1,149 (17.5)	810 (18.2)	117 (19.0)	0.649	184 (15.2)	38 (12.7)	0.282
History of TIA	426 (6.5)	287 (6.5)	41 (6.6)	0.854	75 (6.2)	23 (7.7)	0.342
History of MI	402 (6.1)	277 (6.2)	46 (7.5)	0.242	67 (5.5)	12 (4.0)	0.293
*Comorbid conditions*							
Number of comorbid conditions	2.7 ± 1.6	2.8 ± 1.6	2.8 ± 1.7	0.570**	2.6 ± 1.6	2.4 ± 1.6	0.006**
Chronic heart failure	559 (8.5)	378 (8.5)	74 (12.0)	**0.004**	87 (7.2)	20 (6.7)	0.770
Atrial fibrillation	1,083 (16.5)	783 (17.6)	106 (17.2)	0.797	163 (13.4)	31 (10.4)	0.155
Diabetes mellitus	2,679 (40.7)	1,887 (42.4)	246 (39.9)	0.230	449 (37.0)	97 (32.4)	0.140
Hypercholesterolemia	2,403 (36.5)	1,645 (37.0)	198 (32.1)	**0.018**	463 (38.2)	97 (32.4)	0.066
Dementia	541 (8.2)	374 (8.4)	76 (12.3)	**0.001**	75 (6.2)	16 (5.4)	0.588
Depression	753 (11.4)	502 (11.3)	67 (10.9)	0.754	155 (12.8)	29 (9.7)	0.145
Anxiety disorders	1,977 (30.1)	1,337 (30.1)	184 (29.8)	0.907	394 (32.5)	62 (20.7)	**<0.001**
Parkinson’s disease	280 (4.3)	187 (4.2)	33 (5.3)	0.191	49 (4.0)	11 (3.7)	0.775
Epilepsy	181 (2.8)	121 (2.7)	18 (2.9)	0.778	34 (2.8)	8 (2.7)	0.904
Bronchial asthma/COPD	1,340 (20.4)	882 (19.8)	148 (24.0)	**0.016**	236 (19.5)	74 (24.7)	**0.042**
*ACEI-related characteristics*							
Initially administered ACEI							
Perindopril	2,963 (45.0)	1,954 (43.9)	295 (47.8)	**0.005**	577 (47.6)	137 (45.8)	**0.001**
Lisinopril	367 (5.6)	226 (5.1)	43 (7.0)		66 (5.4)	32 (10.7)	
Ramipril	1,184 (18.0)	803 (18.0)	124 (20.1)		191 (15.7)	66 (22.1)	
Enalapril	102 (1.6)	82 (1.8)	7 (1.1)		12 (1.0)	1 (0.3)	
Spirapril	11 (0.2)	6 (0.1)	2 (0.3)		2 (0.2)	1 (0.3)	
Trandolapril	1,226 (18.6)	883 (19.8)	92 (14.9)		217 (17.9)	34 (11.4)	
Quinapril	569 (8.7)	409 (9.2)	39 (6.3)		99 (8.2)	22 (7.4)	
Imidapril	93 (1.4)	51 (1.1)	9 (1.5)		30 (2.5)	3 (1.0)	
Fosinopril	63 (1.0)	35 (0.8)	6 (1.0)		19 (1.6)	3 (1.0)	
New user of ACEIs^b^	437 (6.6)	200 (4.5)	51 (8.3)	**<0.001**	159 (13.1)	27 (9.0)	0.054
Patient´s co-payment (EUR)^c^	3.7 ± 3.1	3.7 ± 3.1	3.4 ± 2.9	0.727**	3.6 ± 2.9	3.2 ± 2.7	0.374**
General practitioner as index prescriber	5,412 (82.3)	3,754 (84.4)	505 (81.8)	0.107	943 (77.7)	210 (70.2)	**0.006**
*CV co-medication*							
Number of medications	7.9 ± 2.7	8.0 ± 2.6	7.7 ± 2.7	0.013**	7.6 ± 2.9	7.3 ± 2.9	0.037**
Number of CV medications	4.8 ± 2.2	4.9 ± 2.3	4.8 ± 2.2	0.198**	4.7 ± 2.2	4.4 ± 2.0	0.041**
Antiplatelet agents	4,630 (70.4)	3,130 (70.4)	444 (72.0)	0.411	845 (69.7)	211 (70.6)	0.760
Anticoagulants	1,772 (26.9)	1,229 (27.6)	180 (29.2)	0.421	293 (24.2)	70 (23.4)	0.787
Cardiac glycosides	641 (9.7)	466 (10.5)	75 (12.2)	0.205	84 (6.9)	16 (5.4)	0.327
Antiarrhythmic agents	540 (8.2)	378 (8.5)	55 (8.9)	0.728	93 (7.7)	14 (4.7)	0.071
Beta-blockers	1,275 (19.4)	895 (20.1)	116 (18.8)	0.443	228 (18.8)	36 (12.0)	**0.006**
Thiazide diuretics	1,456 (22.1)	1,053 (23.7)	124 (20.1)	**0.049**	228 (18.8)	51 (17.1)	0.487
Loop diuretics	1,583 (24.1)	1,097 (24.7)	182 (29.5)	**0.009**	248 (20.4)	56 (18.7)	0.507
Mineralocorticoid receptor antagonists	553 (8.4)	368 (8.3)	83 (13.5)	**<0.001**	80 (6.6)	22 (7.4)	0.638
Calcium channel blockers	1,871 (28.4)	1,334 (30.0)	149 (24.1)	**0.003**	325 (26.8)	63 (21.1)	**0.042**
Statins	4,394 (66.8)	2,928 (65.8)	367 (59.5)	**0.002**	884 (72.9)	215 (71.9)	0.736
Lipid-lowering agents other than statins^d^	544 (8.3)	372 (8.4)	39 (6.3)	0.082	102 (8.4)	31 (10.4)	0.284

In the case of categorical variables, values represent the frequency and the percentages are provided in parentheses (% of n). In the case of continuous variables, means ± standard deviations are provided. CV, cardiovascular; TIA, transient ischemic attack; MI, myocardial infarction; COPD, chronic obstructive pulmonary disease; ACEI, angiotensin-converting enzyme inhibitor; p, statistical significance between adherent and non-adherent patients according to the χ^2^-test; ** statistical significance according to the Mann-Whitney U test; in the case of statistical significance (*p* < 0.05), the values are expressed in bold.

^a^ The time period covered by “history”—5 years before the index date of this study.

^b^ New user of ACEIs—patient in whom ACEI treatment was initiated in association with the diagnosis of peripheral arterial disease.

^c^ Co-payment—calculated as the cost of ACEI treatment paid by the patient per month.

^d^ Lipid-lowering agents other than statins—ezetimibe and fibrates.

**TABLE 2:** Baseline characteristics of the cohort of ARB users.

Factor	ARB users (n = 502)						
	All ARB users (n = 502)	Persistent (n = 372; 74.1%)			Non-persistent (n = 130; 25.9%)		
		Adherent (n = 322; 86.6%)	Non-adherent (n = 50; 13.4%)	p	Adherent (n = 103; 79.2%)	Non-adherent (n = 27; 20.8%)	p
*Socio-demographic characteristics*							
Age	74.6 ± 6.5	74.4 ± 6.6	76.3 ± 6.7	0.036**	74.2 ± 6.2	75.8 ± 6.4	0.195**
Female sex	302 (60.2)	188 (58.4)	28 (56.0)	0.750	72 (69.9)	14 (51.9)	0.078
University education	23 (4.6)	15 (4.7)	3 (6.0)	0.720*	4 (3.9)	1 (3.7)	1.000*
Employed patients	16 (3.2)	12 (3.7)	3 (6.0)	0.436*	0 (0.0)	1 (3.7)	0.208*
*History of CV events* ^a^							
History of ischemic stroke	89 (17.7)	55 (17.1)	10 (20.0)	0.613	20 (19.4)	4 (14.8)	0.782*
History of TIA	36 (7.2)	20 (6.2)	5 (10.0)	0.357*	9 (8.7)	2 (7.4)	1.000*
History of MI	21 (4.2)	14 (4.3)	2 (4.0)	1.000*	4 (3.9)	1 (3.7)	1.000*
*Comorbid conditions*							
Number of comorbid conditions	2.6 ± 1.6	2.6 ± 1.6	2.7 ± 1.8	0.772**	2.6 ± 1.5	2.0 ± 1.3	0.057**
Chronic heart failure	26 (5.2)	17 (5.3)	4 (8.0)	0.505*	4 (3.9)	1 (3.7)	1.000*
Atrial fibrillation	62 (12.4)	42 (13.0)	11 (22.0)	0.092	8 (7.8)	1 (3.7)	0.684*
Diabetes mellitus	200 (39.8)	129 (40.1)	18 (36.0)	0.585	45 (43.7)	8 (29.6)	0.186
Hypercholesterolemia	186 (37.1)	123 (38.2)	16 (32.0)	0.399	35 (34.0)	12 (44.4)	0.314
Dementia	30 (6.0)	21 (6.5)	4 (8.0)	0.760*	4 (3.9)	1 (3.7)	1.000*
Depression	49 (9.8)	30 (9.3)	6 (12.0)	0.605*	12 (11.7)	1 (3.7)	0.300*
Anxiety disorders	145 (28.9)	94 (29.2)	12 (24.0)	0.449	35 (34.0)	4 (14.8)	0.053
Parkinson’s disease	23 (4.6)	16 (5.0)	1 (2.0)	0.712*	6 (5.8)	0 (0.0)	0.343*
Epilepsy	14 (2.8)	8 (2.5)	3 (6.0)	0.173*	3 (2.9)	0 (0.0)	1.000*
Bronchial asthma/COPD	85 (16.9)	58 (18.0)	8 (16.0)	0.729	17 (16.5)	2 (7.4)	0.360*
*ARB-related characteristics*							
Initially administered ARB							
Valsartan	159 (31.7)	88 (27.3)	17 (34.0)	0.646	42 (40.8)	12 (44.4)	0.168*
Losartan	137 (27.3)	97 (30.1)	16 (32.0)		15 (14.6)	9 (33.3)	
Telmisartan	107 (21.3)	70 (21.7)	10 (20.0)		24 (23.3)	3 (11.1)	
Candesartan	87 (17.3)	59 (18.3)	7 (14.0)		18 (17.5)	3 (11.1)	
Irbesartan	12 (2.4)	8 (2.5)	0 (0.0)		4 (3.9)	0 (0.0)	
New user of ARBs^b^	19 (3.8)	10 (3.1)	4 (8.0)	0.104*	5 (4.9)	0 (0.0)	0.583*
Patient´s co-payment (EUR)^c^	2.3 ± 1.8	2.2 ± 1.7	2.4 ± 1.9	0.520**	2.7 ± 1.9	2.6 ± 1.8	0.773**
General practitioner as index prescriber	409 (81.5)	272 (84.5)	37 (74.0)	0.066	76 (73.8)	24 (88.9)	0.097
*CV co-medication*							
Number of medications	7.8 ± 2.7	7.9 ± 2.6	7.5 ± 2.8	0.338**	7.7 ± 2.8	7.3 ± 2.9	0.487**
Number of CV medications	4.8 ± 2.2	4.8 ± 2.2	4.5 ± 2.3	0.104**	4.9 ± 2.2	4.1 ± 1.4	0.188**
Antiplatelet agents	359 (71.5)	234 (72.7)	35 (70.0)	0.695	72 (69.9)	18 (66.7)	0.746
Anticoagulants	106 (21.1)	68 (21.1)	17 (34.0)	**0.044**	17 (16.5)	4 (14.8)	1.000*
Cardiac glycosides	40 (8.0)	23 (7.1)	9 (18.0)	0.025*	7 (6.8)	1 (3.7)	1.000*
Antiarrhythmic agents	35 (7.0)	25 (7.8)	5 (10.0)	0.577*	5 (4.9)	0 (0.0)	0.583*
Beta-blockers	117 (23.3)	85 (26.4)	9 (18.0)	0.204	17 (16.5)	6 (22.2)	0.571*
Thiazide diuretics	89 (17.7)	54 (16.8)	6 (12.0)	0.394	25 (24.3)	4 (14.8)	0.293
Loop diuretics	79 (15.7)	55 (17.1)	10 (20.0)	0.613	13 (12.6)	1 (3.7)	0.298*
Mineralocorticoid receptor antagonists	20 (4.0)	15 (4.7)	1 (2.0)	0.707*	4 (3.9)	0 (0.0)	0.580*
Calcium channel blockers	176 (35.1)	128 (39.8)	10 (20.0)	**0.007**	32 (31.1)	6 (22.2)	0.368
Statins	327 (65.1)	207 (64.3)	26 (52.0)	0.095	72 (69.9)	22 (81.5)	0.231
Lipid-lowering agents other than statins^d^	45 (9.0)	26 (8.1)	1 (2.0)	0.151*	17 (16.5)	1 (3.7)	0.119*

In the case of categorical variables, values represent the frequency and the percentages are provided in parentheses (% of n). In the case of continuous variables, means ± standard deviations are provided. CV, cardiovascular; TIA, transient ischemic attack; MI, myocardial infarction; COPD, chronic obstructive pulmonary disease; ARB, angiotensin receptor blocker; *p*, statistical significance between adherent and non-adherent patients according to the χ^2^-test; * statistical significance according to the Fisher exact test; ** statistical significance according to the Mann-Whitney *U* test; in the case of statistical significance (*p* < 0.05), the values are expressed in bold.

^a^ The time period covered by “history”—5 years before the index date of this study.

^b^ New user of ARBs—patient in whom ARB treatment was initiated in association with the diagnosis of peripheral arterial disease.

^c^ Co-payment—calculated as the cost of ARB treatment paid by the patient per month.

^d^ Lipid-lowering agents other than statins—ezetimibe and fibrates.

In the group of ACEI users, 5,066 (77.0%) patients were persistent and 1,512 (23.0%) non-persistent with ACEI treatment. Within the group of persistent patients, non-adherence (PDC<80%) was observed in 617 (12.2%) patients, while in the group of non-persistent patients, non-adherence was identified in 299 (19.8%) patients (*p* < 0.001 according to the χ^2^-test). In the group of ARB users, 372 (74.1%) patients were persistent and 130 (25.9%) non-persistent with ARB treatment. Among persistent patients, non-adherence was found in 50 (13.4%) patients, whereas in the group of non-persistent patients, 27 (20.8%) patients were defined as non-adherent (*p* = 0.046 according to the χ^2^-test). Table 1 and Table 2 provide data on socio-demographic characteristics, history of CV events, comorbid conditions, ACEI/ARB related characteristics and CV co-medication in patients of our study cohort.

**FIGURE 1:**
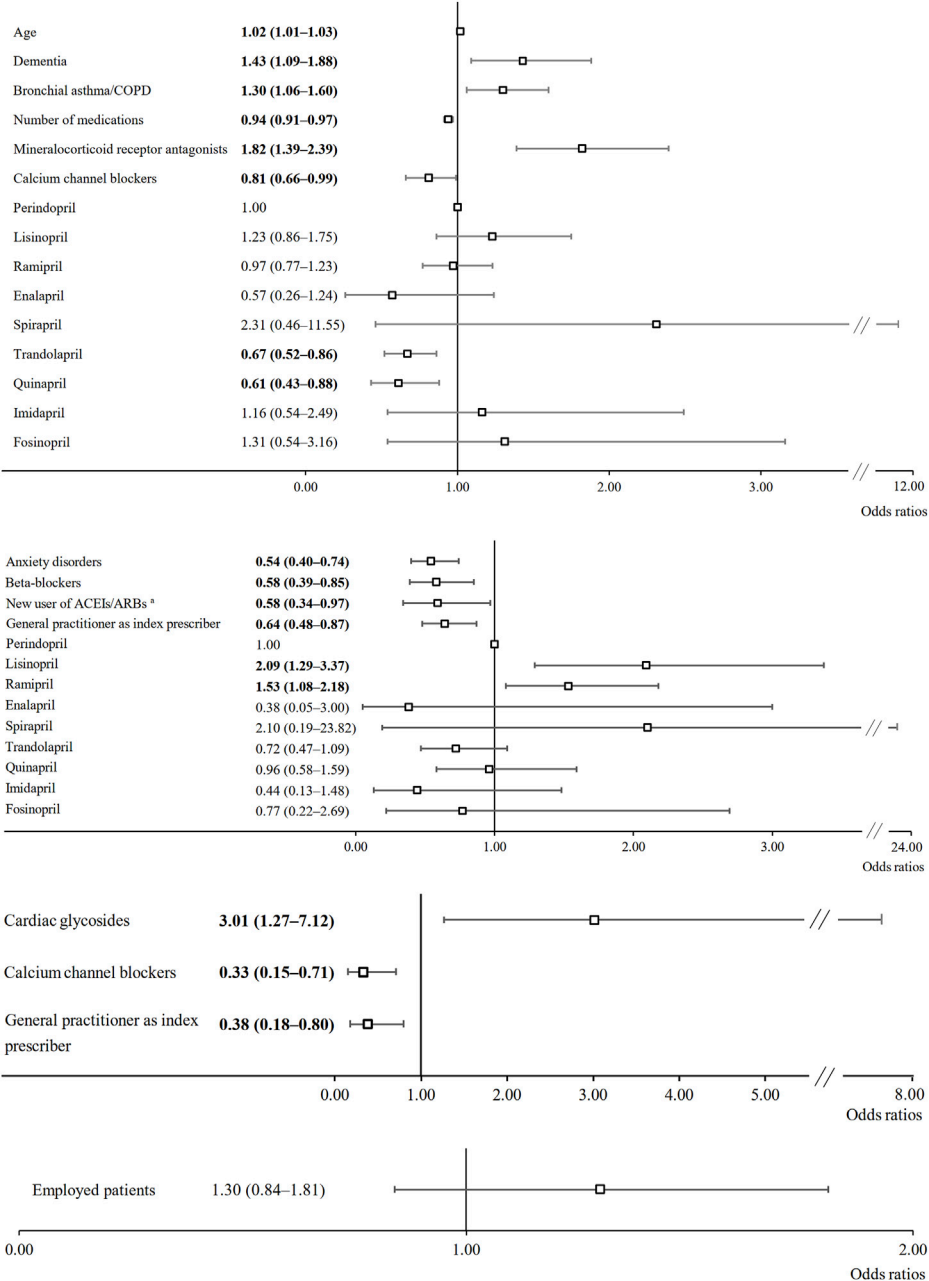
Multivariate analysis of the influence of patient- and medication-related characteristics on the probability of non-adherence; a) persistent ACEI users (n = 5,066); b) non-persistent ACEI users (n = 1,512); c) persistent ARB users (n = 372); c) non-persistent ARB users (n = 130). Values represent odds ratios (95% confidence intervals). In the case of statistical significance (*p* < 0.05), the values are expressed in bold. COPD, chronic obstructive pulmonary disease; ACEI, angiotensin-converting enzyme inhibitor; ARB, angiotensin receptor blocker. ^a^New user of ACEIs/ARBs, patient in whom ACEI/ARB treatment was initiated in association with the diagnosis of peripheral arterial disease.

Factors associated with the likelihood of non-adherence to ACEI/ARB treatment are listed in Figure 1. Among persistent ACEI users, increasing age, dementia, bronchial asthma/chronic obstructive pulmonary disease and administration of mineralocorticoid receptor antagonists were associated with an increased probability of non-adherence, while increasing number of medications, administration of calcium channel blockers, trandolapril and quinapril were associated with adherence. Among non-persistent ACEI users, administration of lisinopril and ramipril were associated with an increased likelihood of non-adherence, while anxiety disorders, being a new user of ACEIs, general practitioner as index prescriber and administration of beta-blockers were associated with adherence. Among persistent ARB users, administration of cardiac glycosides was associated with an increased probability of non-adherence, whereas administration of calcium channel blockers and general practitioner as index prescriber were associated with adherence. In non-persistent ARB users, no factors associated with the probability of non-adherence were found.

### 3.1 Sensitivity analysis

In the case of the model with 3-year follow-up period, the group of ACEI users included 5,228 (79.5%) persistent and 1,350 (20.5%) non-persistent patients of which 13.6% and 18.7% were non-adherent, respectively. Of ARB users, 390 (77.7%) were persistent and 112 (22.3%) non-persistent of which 15.6% and 18.8% were non-adherent, respectively. Supplementary Figure S1 lists factors associated with non-adherence in the case of the model with 3-year follow-up period. Similar characteristics associated with the likelihood of non-adherence were found in this model compared to the main model with 5-year follow-up period. However, there were some differences. Age was not associated with the likelihood of non-adherence in the model with 3-year follow-up period. General practitioner as index prescriber was associated with adherence in non-persistent ACEI users in the main model with 5-year follow-up period, whereas it was associated with adherence in persistent ACEI users in the model with 3-year follow-up period.

## 4 Discussion

No significant difference was found in the overall proportions of non-adherent patients between ACEI and ARB users. Non-adherence was significantly more common among non-persistent patients *versus* persistent patients among both ACEI and ARB users. These results indicate that discontinuation is commonly preceded by decreased adherence (poor implementation). More factors associated with the likelihood of non-adherence were found in the groups of ACEI users compared to ARB users.

### 4.1 Factors associated with the likelihood of non-adherence

Increasing age was associated with an increased likelihood of non-adherence among persistent ACEI users. In our previous manuscript (Wawruch et al., 2022), increasing age represented a protective factor associated with persistence among older women. These results may indicate that in older PAD patients, implementation of ACEI use in their daily regimen is insufficient despite persistence with ACEIs. The design of the present study and of our previous studies does not make it possible to explain these contradictory results. Older patients aged ≥65 years were more likely to be non-adherent to antihypertensive treatment also in a cross-sectional study among Lebanese patients with hypertension (Abbas et al., 2020). On the other hand, older age (≥60 years) increased the probability of adherence to antihypertensive medication in a cross-sectional study in healthcare settings in Islamabad (Mahmood et al., 2020). According to the review of Burnier et al. (2020), medication adherence is better in hypertensive patients aged 65–80 years compared to younger ones. However, in patients aged >80 years, non-adherence increases. Non-adherence in this age group was attributed to specific risk factors like cognitive ability, depression, and health beliefs.

Among comorbid conditions, dementia and bronchial asthma/chronic obstructive pulmonary disease were associated with non-adherence in the group of persistent ACEI users. Dementia decreased the likelihood of non-persistence with ACEI/ARB treatment in our previous study (Wawruch et al., 2022). Poor implementation of ACEI/ARB treatment in this study may be associated with forgetfulness to take medication among patients with cognitive decline. According to the systematic review by Smith et al. (2017), poor cognitive function represents a significant risk factor for non-adherence, and caregivers are therefore important for supporting adherence. Mental comorbidity was positively associated with non-adherence to antihypertensive medication in the study by Calderon-Larranaga et al. (2016). That study analysed the relationship between mental and physical comorbidity and non-adherence to antihypertensive medication among patients attending primary care. Presence of any comorbidity was associated with adherence in a cross-sectional study by Mahmood et al. (2020). However, that study analysed comorbidities as a dichotomous variable (present or not present) without specifying the number and nature of comorbidities.

Anxiety disorders were associated with adherence among non-persistent ACEI users. This result may be associated with meticulous medication-taking behaviour in anxious patients who had regularly taken their medication before discontinuing it. In contrast to our finding, Bautista et al. (2012) concluded that patients with at least mild anxiety are at an increased likelihood of non-adherence to antihypertensive treatment. Their longitudinal cohort study included patients aged 20–70 years who started antihypertensive treatment, had no other chronic comorbid condition, and did not take mood-modifying medications.

Increasing number of medications was associated with adherence in the group of persistent ACEI users. This factor was associated with persistence with ACEI/ARB treatment in our previous study (Wawruch et al., 2022). This result may indicate a careful medication-taking behaviour in patients who are used to take concomitantly several medications. Increasing number of medications was associated with adherence also in a cross-sectional study by Mahmood et al. (2020). According to the authors of that study, better adherence in patients using more than one medication to control their blood pressure may be related to an increased severity of symptoms which forces patients to be adherent to their medications. On the other hand, polypharmacy represented a factor associated with non-adherence to antihypertensive medication in a cross-sectional study by Calderon-Larranaga et al. (2016). Authors of that study did not provide explanation for this finding.

Among CV co-medication, administration of cardiac glycosides was associated with an increased likelihood of non-adherence among persistent ARB users, while mineralocorticoid receptor antagonists increased the probability of non-adherence among persistent ACEI users. In addition, administration of beta-blockers was associated with adherence among non-persistent ACEI users, and administration of calcium channel blockers was associated with adherence among persistent ACEI users and persistent ARB users. In our previous study (Wawruch et al., 2022), administration of beta-blockers and calcium channel blockers was associated with persistence with ACEI/ARB treatment. These results indicate favourable association of beta-blockers and calcium channel blockers with both implementation and persistence phases of adherence. In line with our findings, in a cross-sectional study by Thew et al. (2022), non-usage of calcium channel blockers represented one of four factors associated with non-adherence among patients with uncontrolled hypertension.

Among non-persistent ACEI users, being a new user of ACEIs was associated with adherence to treatment. In our previous study (Wawruch et al., 2022), being a new user of ACEI/ARB treatment was associated with non-persistence. These results indicate that despite insufficient persistence in new users of ACEI/ARB treatment, they seem to take medications properly before discontinuation. According to a systematic review and meta-analysis by Ofori-Asenso et al. (2018), being a new user represented a factor associated with an increased probability of non-adherence to statin treatment among patients aged ≥65 years.

General practitioner as index prescriber was associated with adherence among non-persistent ACEI users and persistent ARB users. In our previous study (Wawruch et al., 2022), general practitioner as index prescriber represented a factor associated with persistence. These results suggest a key role of general practitioners who favourably influence both implementation and persistence phases of adherence. In a study analysing the first-year adherence to antihypertensive therapy among Korean outpatients by Sung et al. (2009), higher likelihood of good adherence was reported in the case when the physician specialised in internal medicine *versus* family medicine or had some other specialisation. Authors of that study did not explain their finding.

Some ACEIs used in our study were associated with the likelihood of non-adherence compared to perindopril. Lisinopril and ramipril were associated with an increased likelihood of non-adherence among non-persistent ACEI users, while trandolapril and quinapril were associated with adherence among persistent ACEI users. In our previous study, administration of imidapril, fosinopril and valsartan was associated with non-persistence, while enalapril was associated with persistence with ACEI/ARB treatment (Wawruch et al., 2022). The design of our study does not make it possible to explain these findings. Cui et al. (2020) analysed adherence to antihypertensive drugs in Chinese patients. Valsartan belonged to drugs with the highest values of Medication Possession Ratio (MPR), while benazepril had the lowest MPR.

### 4.2 Study limitations

Our study has some limitations which should be considered when interpreting the study results. The database of the General Health Insurance Company is created for insurance purposes and not for research. It is impossible to identify whether medications were taken as prescribed and to determine who was responsible for treatment discontinuation (patient or physician). Data on adverse effects were not available in this database. Other limitation consists in the small number of ARB users compared to ACEI users. This significant difference may be explained by the preference of ACEIs as the first-choice drugs and the use of ARBs in the case of intolerance of ACEIs. On the other hand, the large sample size which covers all regions of the Slovak Republic as well as detailed and precise data on patients’ comorbid conditions and medications represent the strengths of our study.

## 5 Conclusion

In our study, no differences in the proportions of non-adherent patients were found between older ACEI and ARB users with PAD. Significantly higher proportions of non-adherent patients were found among non-persistent patients in comparison with persistent patients among both ACEI and ARB users. This result indicates worsening of adherence before discontinuation of treatment among non-persistent patients. Factors associated with non-adherence identified in our study may give indications for identifying patients at an increased probability of non-adherence in whom special attention should be paid to improving their adherence so as to ensure effective secondary prevention of PAD.

## Data Availability

The data that support the findings of this study are available from the General Health Insurance Company but restrictions apply to the availability of these data, which were used under license for the current study, and so are not publicly available. Data are however available from the authors upon reasonable request and subject to permission of the General Health Insurance Company.
