# Supplementary material for: Factors associated with non-adherence to angiotensin-converting enzyme inhibitors and angiotensin receptor blockers in older patients with peripheral arterial disease
Source: Front Pharmacol. 2023 Aug 10;14:1199669. doi: 10.3389/fphar.2023.1199669 (PMC10448819; doi:10.3389/fphar.2023.1199669)
Supplement: Supplementary file 1 [file DataSheet1.PDF]

Supplementary Material

Factors Associated with Non-Adherence to Angiotensin-Converting Enzyme Inhibitors and Angiotensin Receptor Blockers in Older Patients with Peripheral Arterial Disease

Martin Wawruch[1*], Miriam Petrova[1], Tomas Tesar[2*], Jan Murin[3], Patricia Schnorrerova[1], Martina Paduchova[4], Denisa Celovska[3], Beata Havelkova[5], Michal Trnka[6], Sofa D. Alfian[7,8], Emma Aarnio[9]

*** Correspondence:** Martin Wawruch martin.wawruch@gmail.com; Tomas Tesar tesar@fpharm.uniba.sk

Supplementary Figure 1. Multivariate analysis of the influence of patient- and medication-related characteristics on the probability of non-adherence evaluated in the model with a shorter 3-year follow-up period; a) persistent ACEI users (n = 5228); b) non-persistent ACEI users (n = 1350); c) persistent ARB users (n = 390); d) non-persistent ARB users (n = 112).

a)

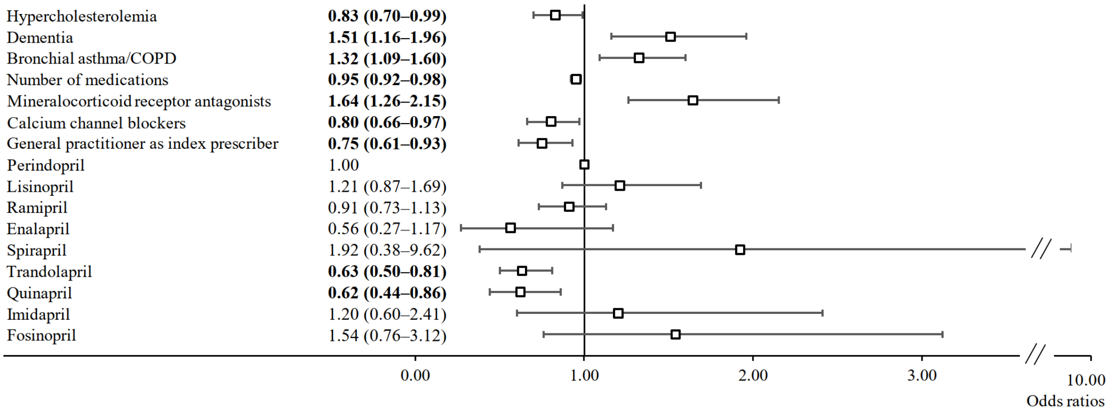

b)

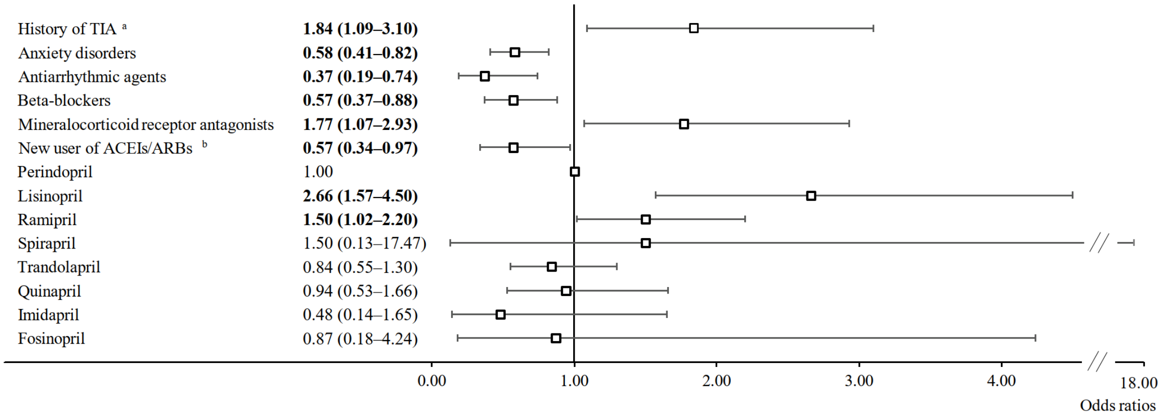

c)

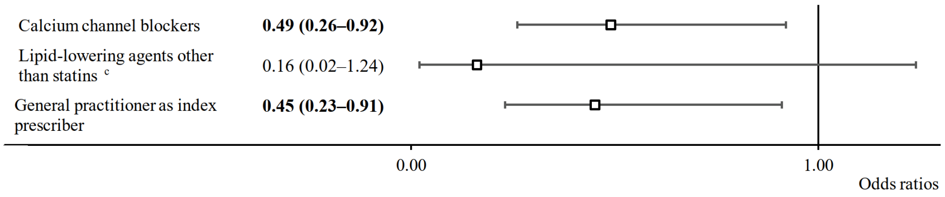

d)

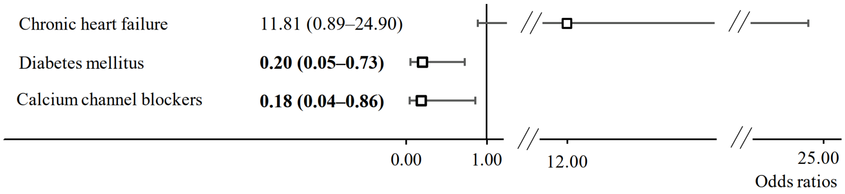

Values represent odds ratios (95% confidence intervals). In the case of statistical significance ($p<0.05$), the values are expressed in bold. COPD – chronic obstructive pulmonary disease; TIA – transient ischemic attack; ACEI – angiotensin-converting enzyme inhibitor; ARB – angiotensin receptor blocker. [a]The time period covered by “history” – 5 years before the index date of this study. [b]New user of ACEIs/ARBs – patient in whom ACEI/ARB treatment was initiated in association with the diagnosis of peripheral arterial disease. [c]Lipid-lowering agents other than statins – ezetimibe and fibrates.
